# Stable and transient transformation, and a promoter assay in the selective lignin-degrading fungus, *Ceriporiopsis subvermispora*

**DOI:** 10.1186/s13568-019-0818-1

**Published:** 2019-06-24

**Authors:** Yoichi Honda, Eiji Tanigawa, Takahisa Tsukihara, Dong Xuan Nguyen, Harunori Kawabe, Naofumi Sakatoku, Junko Watari, Hideaki Sato, Shigekazu Yano, Takashi Tachiki, Toshikazu Irie, Takahito Watanabe, Takashi Watanabe

**Affiliations:** 1 Graduate School of Agriculture, Kyoto University, Kyoto, 606-8502 Japan; 2 Research Institute for Sustainable Humanosphere, Kyoto University, Uji, 611-0011 Japan; 3 Biotechnology Center of Ho Chi Minh City, Ho Chi Minh City, Vietnam; 4 Graduate School of Life Sciences, Ritsumeikan University, Kusatsu, 525-8577 Japan; 5 Environmental Science Graduate School, The University of Shiga Prefecture, Hikone, 522-0057 Japan; 6 Present Address: Graduate School of Science and Engineering, Yamagata University, Yonezawa, 992-8510 Japan

**Keywords:** Basidiomycete, Genetic transformation, Transient gene expression, Promoter assay, TATAA sequence

## Abstract

A genetic transformation system was developed for the selective white rot basidiomycete *Ceriporiopsis subvermispora* using a modified protocol with polyethylene glycol and CaCl_2_ treatment of the protoplasts and plasmids harboring recombinant hygromycin phosphotransferase (*hph*) driven by a homologous promoter. During repeated transfer on fresh potato dextrose agar plates containing 100 µg/ml hygromycin B, most transformants lost drug resistance, while the remaining isolates showed stable resistance over five transfers. No drug-resistant colonies appeared in control experiments without DNA or using a promoter-less derivative of the plasmid, indicating that a transient expression of the recombinant *hph* was driven by the promoter sequence in these unstable drug-resistant transformants. Southern blot analysis of the stable transformants revealed random integration of the plasmid DNA fragment in the chromosome at different copy numbers. This transformation system yielding mostly transient transformants was successfully used for promoter assay experiments, and only a 141-bp fragment was found to be essential for the basic promoter function of glyceraldehyde dehydrogenase gene (*gpd*) in this fungus. Subsequent mutational analyses suggested that a TATAA sequence is important for the basic promoter function of *gpd* gene. The promoter assay system will enable the functional analysis of gene expression control sequences quickly and easily, mostly in the absence of undesirable effects from differences in copy number and chromosomal position of an integrated reporter gene among stable transformants.

## Introduction

Genetic transformation is a powerful tool to investigate the function of a gene of interest and to perform molecular breeding of new strains with desired properties in a specific organism. The development of a genetic transformation system in filamentous basidiomycetes was first reported in model species like *Schizophyllum commune* (Munoz-Rivas et al. 1986) and *Coprinopsis cinerea* (Binninger et al. 1987), followed by commercially important mushrooms such as *Lentinula edodes* (Sato et al. 1998), *Pleurotus ostreatus* (Peng et al. 1992; Honda et al. 2000), *Agaricus bisporus* (Van de Rhee et al. 1996), *Flammulina velutipes* (Kuo et al. 2004), and *Ganoderma lucidum* (Sun et al. 2001). Recently, a transformation system was developed in *Agrocybe aegerita* (Herzog et al. 2019) and *Pleurotus eryngii* (Shang et al. 2018). However, transformation remains a challenge in many basidiomycete fungi, possibly because of the absence of a compatible marker gene, the incompatibility of many gene expression signals, the low efficiency of DNA fragment introduction into the host chromosome, and/or the inefficient protoplast production/regeneration rate. However, because genetic transformation is required as a fundamental platform for genome editing techniques such as CRISPR/Cas9 (Shi et al. 2017), a protocol is essential to promote molecular genetics in species of specific research interest.

The transient expression of an introduced gene in an extrachromosomal manner has been widely recognized in animal cells and plant protoplasts, and is known as ‘transfection’. However, in filamentous fungi, studies have mainly focused on the stable expression of genes integrated in the host chromosome, except for gene expression by extrachromosomal plasmids with an autonomously replicating DNA sequence, *AMA1* (Gems et al. 1991). In an integrated transformation, a transformant is expected to maintain and express the introduced gene stably during mitotic and meiotic cell divisions; thus, in most studies, a stable transformation is the goal in molecular breeding to produce a strain with a desired phenotype suitable for either basic research or industrial applications. Generally, ectopic integrations occur at random sites of the host chromosome with different copy numbers, which makes it difficult to use stable transformants in an assay of gene expression control sequence in filamentous fungi. Although gene targeting or genome editing may be used for assay of a gene expression control sequence, these technologies can be applicable in a limited number of model mushrooms and it is laborious and time-consuming to obtain strains with a series of mutations in the target region.

White rot fungi belong to the *Basidiomycetes* and are important research materials in industrial applications such as the pretreatment of lignocellulose in biorefinery (Sawada et al. 1995; Chandel et al. 2015) or the bioremediation of polluted environments (Fitzgibbon et al. 1995; Kües 2015; Treu and Falandysz 2017). Transformation systems have been developed for some white rot fungi, like *P. ostreatus* (Peng et al. 1992; Honda et al. 2000) and *Phanerochaete chrysosporium* (Alic et al. 1989), and have been used for the functional analysis of genes related to plant biomass degradation (Irie et al. 2001b; Tsukihara et al. 2008; Salame et al. 2012; Nakazawa et al. 2017).

*Ceriporiopsis subvermispora* is a selective white rot fungus (Otjen et al. 1987) because it decomposes plant cell wall lignin without the concomitant severe degradation of cellulose (Messner and Srebotnik 1994). It was screened for biopulping (Wall et al. 1993) and the effective pretreatment of lignocellulosic materials to be saccharified and converted to various useful compounds via fermentation (Itoh et al. 2003; Amirta et al. 2006; Sasaki et al. 2011). It has also been used in the biological treatment of plant biomass to make the substrate more digestible as animal feed (Okano et al. 2005; Nayan et al. 2018). The mechanism of selective wood degradation has been investigated in biochemical and enzymatical studies (Urzúa et al. 1998; Watanabe et al. 2002; Ohashi et al. 2007; Nishimura et al. 2012), and genes encoding extracellular enzymes thought to be responsible for lignin degradation have been cloned and their expression characterized (Tello et al. 2000; Manubens et al. 2003). Furthermore, whole genome sequences were determined and subjected to comparative genomics studies (Fernandez-Fueyo et al. 2012). However, no transformation protocol has been developed in this fungus despite its high potential in industrial applications and continuous trials in many research groups.

In this study, we developed a genetic transformation system in *C. subvermispora* for the first time, using newly constructed recombinant plasmids carrying bacterial hygromycin phosphotransferase (*hph*) gene driven by a homologous promoter sequence from glyceraldehyde dehydrogenase (*gpd*) or ß-tubulin (*ß*-*tub*) genes. We found that stable and unstable drug-resistant transformants were obtained on the screening plate. This occurred in a promoter-dependent manner, and we successfully utilized a promoter assay of homologous *gpd* to determine the minimal essential sequences required for gene expression.

## Materials and methods

### Strains and plasmids

*Ceriporiopsis subvermispora*, Fp-90031-sp (ATCC 90467) was used as a host strain for the introduction of DNA. Strains were grown on potato dextrose agar (PDA) agar plates at 28 °C for maintenance. *Escherichia coli* JM109 [*recA1 endA1 gyrA96 thi hsdR17 supE44 relA1* (∆*lac*-*proAB*)/F (*traD36 proAB lacI*^q^ *lacZ*∆*M15*)] (Yanisch-Perron et al. 1985) was used as a host bacterium for standard recombinant DNA constructions and was grown on Luria–Bertani medium.

**Fig. 1:**
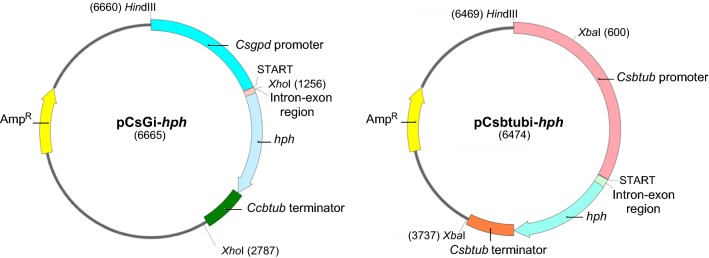
Schematic of pCsGi-*hph* and pCsbtubi-*hph* plasmids. Plasmids pCsGi-*hph* and pCsbtubi-*hph* were constructed by combining the homologous promoter region of *gpd* and beta-tubulin genes from *C. subvermispora*, respectively, with the coding sequence of bacterial *hph*. In pCsGi-*hph*, the first exon, ATGCCC, and the first intron from *Csgpd* were inserted just before the hygromycin phosphotransferase sequence. In pCsbtubi-*hph*, the first and second introns, as well as the first exon (encoding 11 amino acids), from *Csß*-*tub* were inserted just before the hygromycin phosphotransferase sequence. The plasmid backbones of pCsGi-*hph* and pCsbtubi-*hph* are pCRII (Thermo Fisher Scientific Inc.) and pUC19 (Takara Bio Inc.), respectively

Plasmids pLG-*hph* with *hph* under the direction of the *L. edodes gpd* promoter (Hirano et al. 2003) and pPHT1 with *hph* driven by the *ß*-*tubulin* promoter from *C. cinerea* (Cummings et al. 1999) were kindly gifted by Dr. Sato (Iwate Biotechnology Institute, Iwate, Japan) and Prof. Miriam E. Zolan (Indiana University), respectively, and used in transformation experiments. Two new plasmids, pCsGi-*hph* and pCsbtubi-*hph*, containing the coding sequence of bacterial *hph* under the control of the homologous promoter sequence of *gpd* and *ß*-*tub* isolated form *C. subvermispora* were also constructed (Fig. 1). The coding sequence of *hph* was derived from plasmid pPHT1. A deletion series harboring the truncated *gpd* promoter sequence was constructed using the Deletion Kit for Kilo-Sequencing (TaKaRa Biomed, Kyoto, Japan). The mutant plasmid p201[A60C], containing a base substitution from A to C at − 60 nt from the first ATG, was constructed using inverse PCR followed by phosphorylation and ligation of both DNA ends.

### Genetic transformation and promoter assay

The transformation protocol was developed by modifying conditions and media of the PEG/CaCl_2_ method developed for *P. ostreatus.* (Honda et al. 2000). For the cultivation of mycelia for protoplast generation, yeast malt peptone glucose (YMPG) medium (0.2% [wt/vol] yeast extract, 1% [wt/vol] malt extract, 0.2% [wt/vol] tryptone, 1% [wt/vol] glucose, 0.1% [wt/vol] l-asparagine monohydrate, 0.1% [wt/vol] MgSO_4_·7H_2_O, 0.2% [wt/vol] KH_2_PO_4_) was used. A cell wall-lysing enzyme was prepared by ammonium sulfate precipitation of 1 L of culture filtrate of *Trichoderma harzianum* (NBRC 3016) grown on medium containing 0.05% [wt/vol] yeast extract, 0.02% [wt/vol] MgSO_4_·7H_2_O, 0.05% [wt/vol] K_2_HPO_4_, 0.05% [wt/vol] KH_2_PO_4_ and 0.5% [wt/vol] cell wall preparation of *P. eryngii* (Yano et al. 2003) in a 2-L Sakaguchi flask. The precipitation was dissolved in 10 mM citrate buffer (pH 5.0) followed by dialysis and freeze-dry treatment. This enzyme, as well as other commercially obtained enzymes (described below), was used to make protoplasts from cultured mycelium. Different concentrations of PEG and CaCl_2_ were used to compare transformation efficiencies, and optimal concentrations were used thereafter. The first screening used YMPG plates containing 1.5% agar, 1.0 M glucitol (d-sorbitol) as an osmotic stabilizer, and 100 µg/ml hygromycin B. PDA plates with the same concentration of hygromycin B were used in subsequent repeated screening steps. Each colony was transferred on the edge of the secondary screening plates (35-mm diameter) with hygromycin B using an inoculating loop and incubated at 28 °C for several days. When mycelium grew to the opposite end, a plug was punched out from the new edge with a 4-mm-diameter cork borer, followed by transfer to the third screening plates. The same transfer method was repeated for the further screening steps.

The finalized transformation protocol for the promoter assay was as follows: fungal mycelia were pre-cultured on a PDA plate at 28 °C for 7 days. Six pieces of the edge of fresh mycelium were cut out using a cork borer (5-mm diameter), then transferred into each of two 200-ml Erlenmeyer flasks containing 80 ml YMPG liquid media, and incubated at 28 °C for 7–10 days. The cultures were homogenized two to four times (10 s each time) at low speed using a Waring blender. Four ml of fungal suspension were transferred into each of 14 300-ml Erlenmeyer flasks containing 160 ml YMPG media, and incubated at 28 °C for 4–5 days. Fungal mycelia were collected using a draining filter, then washed with MM buffer (0.5 M mannitol, 25 mM maleic acid buffer, pH 5.5). The mycelia mass was poured into a 15-ml tube containing 4 ml enzyme solution (10 mg lysing enzyme from *T. harzianum*, 20 mg zymolyase [Seikagaku, Tokyo, Japan], and 4 mg chitinase [Sigma Aldrich, St Louis, MO] in 4 ml MM buffer) and constantly agitated at 60 rpm at 28 °C for 3 h. Protoplasts were collected by filtering the mixture (filter pore size: 100 µm). The solution was centrifuged at 1019×*g* at 20 °C for 10 min to precipitate protoplasts. The protoplasts were then washed in 4 ml MM buffer and precipitated again by centrifuging at 1019×*g* at 20 °C for 10 min. They were suspended in 5 ml MMC buffer (0.5 M mannitol, 25 mM maleic acid buffer, pH 5.5, 12.5 mM CaCl_2_) and the mixture was centrifuged at 1019×*g* at 20 °C for 10 min. Finally, the protoplasts were suspended in 1 ml MMC buffer and adjusted to a final concentration of ~ 2 × 10^7^ cells/ml.

For fungal transformation, 50 µl of protoplasts were mixed with 1 µl transforming DNA (1.0 µg/µl) in a 1.5-ml tube, then 12.5 µl PEG buffer (30% polyethylene glycol #4000, 10 mM Tris–HCl pH 7.5, 300 mM CaCl_2_) was added and mixed well by gently inverting. After incubation on ice for 15 min, 330 µl PEG buffer was added, mixed, and placed at room temperate for 5 min. Subsequently, 660 µl GTC buffer (2 M glucitol, 10 mM Tris–HCl pH 7.5, 25 mM CaCl_2_) was added to the transformation solution, mixed gently, and finally spread onto YMPG regeneration plates (YMPG medium, 2% [wt/vol] agar, 1.0 M glucitol) containing 200 µg/ml hygromycin B and incubated at 28 °C. The number of hygromycin-resistant transformants was counted after 5 days of incubation.

### Detection of introduced DNA

To detect introduced DNA, Southern blotting and specific amplification of *hph* sequences were performed using genomic DNA extracted from mycelia of hygromycin-resistant isolates and wild-type strain as a control. Southern hybridization was carried out using 1 µg of genomic DNA for each lane. For preparation of genomic DNA, mycelium from the third screening plate without hygromycin was inoculated on a 90-mm-diameter PDA plate using an inoculation loop. After incubation for 1 week, mycelium was collected and inoculated on 100-ml YMPG liquid medium in 300-ml Erlenmeyer flask followed by incubation constantly agitated at 130 rpm at 28 °C for 2 weeks. Genome DNA was extracted by the method described by Honda et al. (2000) Probe DNA was labeled using the DIG DNA labeling kit (Sigma-Aldrich) according to the manufacturer’s instructions using the DNA pol I Klenow fragment, random hexamer primers, and DNA template amplified by conventional PCR with primers listed in Table 1. For genomic PCR of *hph*, 12.5 pmol of each specific primer was added to 0.5 µg of template DNA. For small scale DNA preparation, mycelia were collected from culture on the second screening plate and inoculated on 5 ml of YMPG liquid medium in test tube. After incubation at 28 °C for 1 week, DNA was extracted using the protocol described above, The amplification program was as follows: 98 °C for 1 min, followed by 35 cycles of 98 °C for 30 s, 56 °C for 30 s, and 72 °C for 1 min. After amplification, a 10-μl aliquot of each PCR product was analyzed by 1.0% agarose gel electrophoresis.

**Table 1:** Primers used in this study

Primer name	Sequence (5′-3′)	Purpose
hph-1	ATGAAAAAGCCTGAACTCACCGCGACG	Probe in Southern blot and genomic PCR for pCsGi-*hph*
hph-2	CTATTCCTTTGCCCTCGGACGAGTGCT	Probe in Southern blot and genomic PCR for pCsGi-*hph*
Csbar_F	TCAGGTGCGTGAAGTGTACT	Probe in Southern blot and genomic PCR for pCsbtubi-*hph*
hph-4_R	TACGTAGGTAGTGGTAGACT	Probe in Southern blot and genomic PCR for pCsbtubi-*hph*
CsGPD-TATAmut_R	GAGGGTTGATATGGGGCGAT	Site-directed mutagenesis for p201[A60C]
CsGPD-8	ACCCCGCCGCTTTTTCCCTTCAT	Site-directed mutagenesis for p201[A60C]
CsGP-PRO5	CGCCAAGCTTACTCATCCAGAATACACTCG	PCR amplification of *Csgpd* promoter, and the first exon and intron
CsGP-PRO3int	CTGCAATTGTCCGTCAGTAC	PCR amplification of *Csgpd* promoter, and the first exon and intron
Csbtub-pro-R1	GCTAGAGACGATATCAGTT	PCR amplification of *Csbtub* promoter
Csbtub-Hind_F2	AGGCTCTAAGCTTGTACCGAGTGCAT	PCR amplification of *Csbtub* promoter

## Results

### Stable and unstable transformation in *C. subvermispora*

We tested several plasmids containing a heterologous hygromycin-resistant marker gene to transform *C. subvermispora* protoplasts using a conventional PEG/CaCl_2_ protocol developed for *P. ostreatus* (Honda et al. 2000) and *C. cinerea* (Binninger et al. 1987). These plasmids included pLG-*hph* and pPHT1, which had been successfully utilized in the transformation of *P. ostreatus* (Irie et al. 2001a) and *C. cinerea* (Cummings et al. 1999), respectively. However, in *C. subvermispora*, almost no positive transformants were obtained in repeated experiments (data not shown). The regeneration rate of *C. subvermispora* protoplasts was less than 0.1%, while that of *P. ostreatus* was more than 2%.

In conventional transformation protocols for *P. ostreatus* and *C. cinerea*, sucrose is used as an osmotic stabilizer. Therefore, we next tested several different osmotic stabilizers in the buffer and regenerating plates, including glucitol (d-sorbitol) and MgSO_4_ to obtain higher protoplast regeneration efficiencies. The regeneration efficiency was relatively high (0.8%) when 1.0 M glucitol was used as an osmotic stabilizer (data not shown). Although a relatively high regeneration efficiency was observed using MgSO_4_, this reduced the sensitivity of regenerating protoplasts to hygromycin B. For this reason, 1.0 M glucitol was used in the following experiments.

Next, we constructed new plasmids, pCsGi-*hph* and pCsbtubi-*hph*, harboring the *hph* coding sequence under the control of homologous *gpd* and *ß*-*tub* promoters from *C. subvermispora*, respectively, fused to the *C. subvermispora ß*-*tub* terminator sequence (Fig. 1). With 1.0 M sorbitol as an osmotic stabilizer, pCsGi-*hph* and pCsbtubi-*hph*, as well as pLG-*hph*, were introduced into *C. subvermispora* protoplasts in the presence of conventional concentrations of PEG and CaCl_2_ (25% PEG and 25 mM CaCl_2_). Small numbers of hygromycin-resistant colonies appeared on the screening plate with 100 µg/ml hygromycin B, but they did not continue to grow on the fresh screening plate containing the same concentration of hygromycin B (data not shown).

**Fig. 2:**
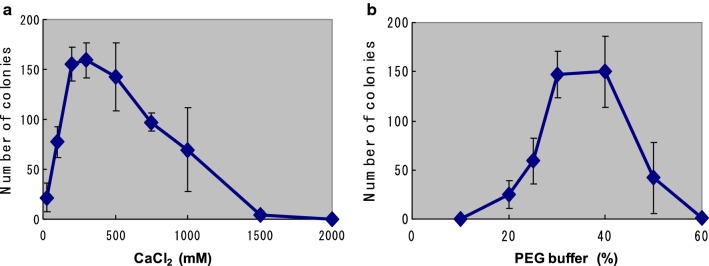
Optimization of conditions for transformation in *C. subvermispora*. Number of drug-resistant colonies on the first screening plate containing 100 µg/ml hygromycin B (n = 3, ± SD) with various concentrations of CaCl_2_ in the presence of 40% PEG (**a**) and various concentrations of PEG in the presence of 300 mM CaCl_2_ (**b**)

To improve the efficiency of transformation, conditions for protoplast treatment were reviewed and different concentrations of PEG and CaCl_2_ were tested (Fig. 2). Using 40% PEG and 200 mM CaCl_2_, plenty of hygromycin-resistant colonies with 2–4-mm diameter were obtained after 5–7 days incubation on regeneration medium containing 100 µg/ml hygromycin B, And some colonies were randomly selected and subjected to repeated screenings on PDA plates containing the same amount of hygromycin B (Table 2). In the three independent experiments described in Table 2, for plasmids pCsGi-*hph* and pCsbtubi-*hph*, 9/90 and 13/120 of HygB-resistant isolates were stably maintained under drug selection and were transferred more than four times, respectively. However, the remaining isolates lost their drug resistance during 1–3 transfers on fresh selection medium in the following repeated screening processes. We have repeated similar experiments and, totally, out of 430 initial drug resistant colonies obtained with pCsGi-*hph*, 158 (36.7%) isolates and 29 (6.7%) isolates showed drug resistance on the 2nd and 3rd screening plates, respectively. With pCsbtubi-*hph*, out of 97 initial hygromycin-resistant colonies, 55 (56.7%) and 9 (9.2%) isolates showed drug resistance on the 2nd and 3rd screening plates, respectively. All the 38 strains grew on the 3rd screening plates showed stable drug resistance in the further repeated screening steps. On the other hand, for the heterologous promoter plasmid, pLG-*hph*, no stable transformants were observed out of a total of 58 isolates (Table 2).

**Table 2:** Transformation efficiencies and stability of drug resistance during repeated screenings

Exp.	Plasmid	Total number of Hyg-resistant colonies in the first screening	Number of Hyg-resistant strains/isolates subjected to the repeated screenings		
			2nd	3rd	4th
1	pCsGi-*hph*	125	6/30	0/6	Not done
	pCsbtubi-*hph*	160	9/40	2/9	2/2
	pLG-*hph*	13	0/13	Not done	Not done
2	pCsGi-*hph*	91	14/30	2/14	2/2
	pCsbtubi-*hph*	137	11/40	4/11	4/4
	pLG-*hph*	20	0/20	Not done	Not done
3	pCsGi-*hph*	157	23/30	7/23	7/7
	pCsbtubi-*hph*	170	19/40	7/19	7/7
	pLG-*hph*	25	0/25	Not done	Not done

The number of colonies on the screening plates with 100 µg/ml of hygromycin B was counted

Similar unstable drug resistant colonies were reported and described as ‘false positives’ or ‘background’ in other filamentous fungi (Peng et al. 1992; Irie et al. 2001b; Kim et al. 2003; Gang et al. 2006). To determine whether they are dependent on transforming plasmid or not, we carried out a no-DNA control experiment and observed no colonies on the screening plate containing 100 µg/ml of hygromycin B. These results demonstrated that the isolates with unstable drug-resistance were not background-colonies of wild-type strain that eluded the screening process nor mutant strains with acquired drug resistance. They might be described as a ‘false positive’ transformant, in such an experiment just aiming for stable transformants as an expected goal. However, in the present study, it was clearly indicated that their transient drug resistance was dependent on the recombinant plasmids harboring the *hph* coding region. Thus, the introduced marker gene was suggested to have been expressed at a sufficient level to permit mycelial growth and to form a colony on the first screening plate containing 100 µg/ml of HygB, after which the expression level reduced to sustain continuous growth during repetitive transfer on fresh screening plates.

### Fate of the DNA fragment introduced in transformants

**Fig. 3:**
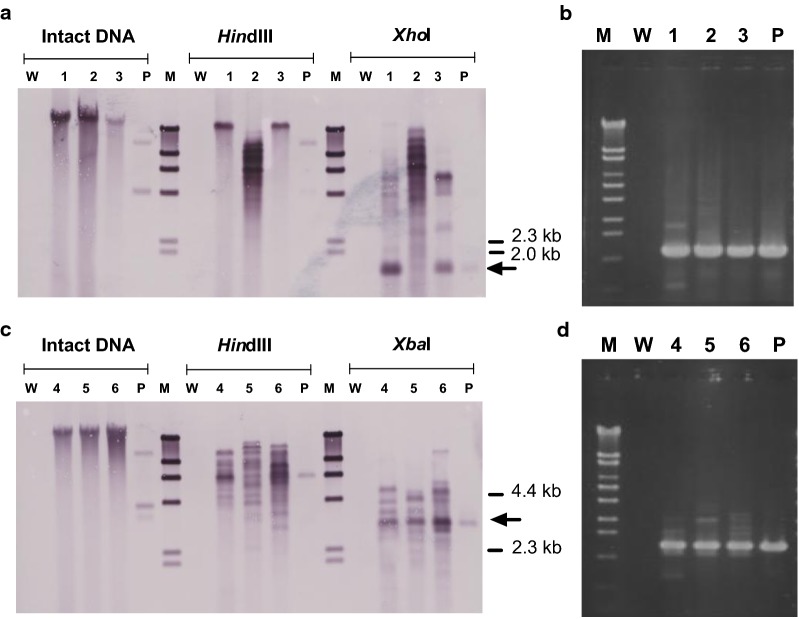
Detection of the *hph* cassette in stable transformants treated with pCsGi-*hph* (**a** and **b**) and pCsbtubi-*hph* (**c** and **d**). **a**, **c** Southern hybridization analysis. Intact DNA, *Hin*dIII- and *Xho*I-digested total genomic DNA from independent transformants (lanes 1–3, pCsGi-*hph*; 4–6, pCsbtubi-*hph*), host wild-type strain (lane W), and the respective plasmid (lane P) were probed with DIG-labeled *hph* sequence. **b**, **d** Specific amplification of *hph* in genomic PCR. Lane M, DNA marker; Lane W, wild-type host strain as a negative control; Lanes 1–3 and 4–6, pCsGi-*hph* and pCsbtubi-*hph* transformants, respectively; Lane P: plasmid pCsGi-*hph* (**b**) and pCsbtubi-*hph* (**d**) as a positive control

Southern hybridization was performed to check the introduced DNA in stable transformants. *hph* was shown to be randomly inserted in high molecular weight DNA at different copy numbers among transformants (Fig. 3a, c). No signal was detected for the wild-type control. Existence of the *hph* sequence in stable transformants was also demonstrated by PCR amplification (Fig. 3b, d). This suggested that stable transformation had occurred by ectopic integration of the vector sequence in the host chromosome, as reported in other basidiomycetes (Munoz-Rivas et al. 1986; Binninger et al. 1987; Honda et al. 2000; Ma et al. 2003).

Similar experiments were conducted for unstable transformants, using genome DNA extracted from mycelium cultivated on non-selective liquid medium. In Southern hybridization and PCR amplification of the recombinant *hph*, a faint signal was detected occasionally (data not shown), suggesting unstable, or very rare, existence of the introduced DNA in these unstable transformants. It is plausible that no chromosomal integration occurred in these unstable transformants, and that the introduced sequence was expressed, most probably, in an extrachromosomal manner, resulting in loss of the introduced sequence during repeated cultivation. However, we cannot rule out the possibility of excision of once-integrated recombinant *hph* on the host chromosome at a very low copy number, which conferred a transient drug resistance in some of the transformants.

### Utilization of the transformation system in determining the minimal sequence of the *gpd* promoter

Table 2 shows that the number of hygromycin-resistant colonies on the first screening plate differed according to the plasmid used. It is plausible that each plasmid has its own transformation efficiency, reflecting the expression level of its recombinant *hph*. As the introduced marker gene is transiently expressed, its transcription should be driven by the promoter sequence. This can be roughly evaluated by the number of the HygB-resistant transformants on the first screening plate, although these may contain a small number of stable transformants.

**Fig. 4:**
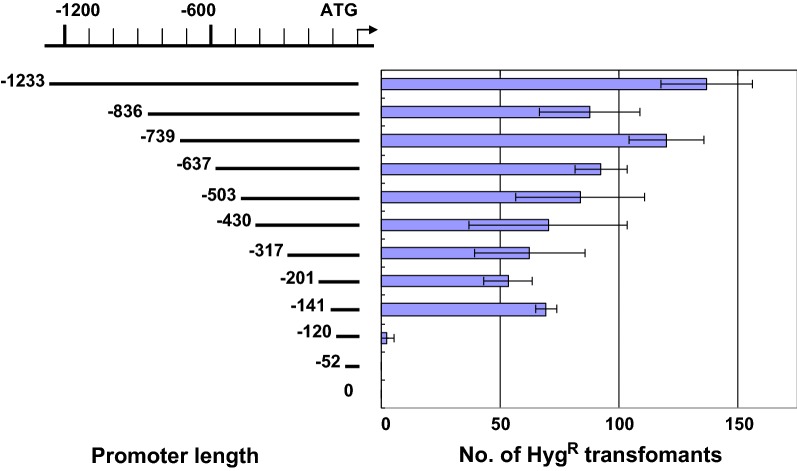
Promoter analysis of *C. subvermispora gpd* using transformants. Promoter constructs used in deletion analysis of the *Csgpd* promoter, and number of drug-resistant transformants (n = 3, ± SD) grown on the first screening plate containing 200 µg/ml hygromycin B. The A nucleotide in the first ATG was counted as +1

To this end, we constructed a series of deletion mutants of the *gpd* promoter region in pCsGi-*hph* and introduced each construct to protoplasts using the slightly modified protocol described above. Here, 30% PEG and 300 mM CaCl_2_ concentrations were used in PEG buffer because this resulted in the optimal transformation efficiency. To make the screening more strict, 200 µg/ml of HygB was used for screening. The number of drug-resistant colonies decreased in line with a decrease in the length of the promoter sequence (Fig. 4). Almost no drug-resistant isolates were obtained when the promoter sequence was shorter than 141 bp. These results indicated that the drug resistance was dependent on the *gpd* promoter, and that the basic promoter function is located within the 141-bp sequence.

### The TATA sequence point mutation reduced *gpd* promoter function

**Fig. 5:**
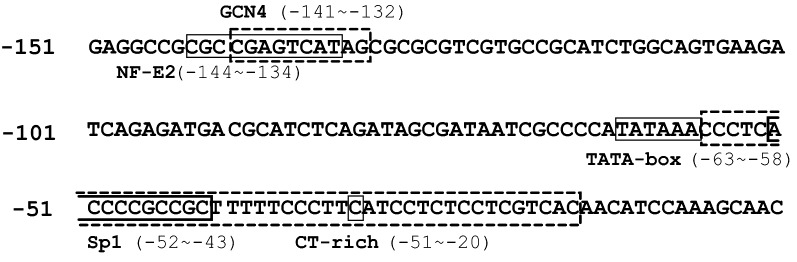
Proposed transcriptional regulation sites in the minimal promoter region of *Csgpd*. Conserved motives mostly characterized in *S. cerevisiae* were found by an in silico motif search. The boxed C nucleotide at position − 36 represents a proposed transcriptional start site predicted from the EST database

We next analyzed cis-acting elements in the 141-bp *C. subvermispora gpd* minimal promoter region. Within this region is a typical TATA box, TATAAA (− 63 to − 58), where A in the first ATG is counted as + 1 (Fig. 5 ). There is also a CT-rich sequence (− 57 to − 20) between the TATA and the first ATG. In ascomycetes, these sequences were demonstrated to be general cis-elements for the promoter region (Punt et al. 1990; Kinghorn and Turner 1992; Lubliner et al. 2015), but little experimental data exist for basidiomycetes. To determine whether the TATAAA (− 63 to − 58) sequence plays an important role in basic promoter function, we introduced a base substitution of C for A (− 60) in plasmid p201, carrying a 201-bp promoter sequence (− 201 to − 1), to produce the mutant plasmid p201[A60C]. Plasmids p201 and p201[A60C] were introduced to the protoplasts of *C. subvermispora* and transformation efficiencies were compared. The average numbers of hygromycin-resistant colonies on the first screening plate for p201 and p201[A60C] were 57.0 and 19.7, respectively, in three independent experiments. This indicated that the point mutation resulted in an approximate threefold reduction in the number of hygromycin-resistant transformants compared to the wild type TATA sequence, showing that it significantly reduced the transforming ability of the plasmid. Moreover, the TATA sequence was confirmed to play an important role in the promoter function.

## Discussion

Unstable drug-resistant transformants have occasionally been reported in the transformation of filamentous fungi (Peng et al. 1992; Schillberg et al. 2000; Irie et al. 2001b; Kim et al. 2003; Gang et al. 2006). In some cases, it was reported that the plasmid was rescued in *E. coli* (Herzog et al. 1995; Randall and Reddy 1992). However, in most cases, these unstable transformants were ignored, or thought of as inert or false positive, because stable transformants have been the focus of interest in view of molecular breeding and functional analysis of gene products. In the present work, we focused on unstable hygromycin resistance in *C. subvermispora* transformation and clearly demonstrated that this phenotype was conferred under the direction of the recombinant *hph* promoter sequence.

Promoter assays are difficult to perform in filamentous fungi because introduced genes are usually randomly integrated in the host chromosome with different copy numbers and at different positions among stable transformants (Mellon and Casselton 1988; Tsukihara et al. 2006; Kilaru et al. 2006). The promoter assay system developed in this work enables the estimation of promoter function largely without undesirable effects from the reporter gene position and copy number on the host chromosome. Although extra-chromosomally replicating plasmids containing an autonomously replicating sequence like *AMA1* were reported in ascomycetes (Gems et al. 1991), these plasmids only function in a limited number of species and did not function in basidiomycetes like *P. ostreatus* (unpublished data). This hampers research progress into the mechanisms of fundamental transcriptional initiation and the transcriptional regulation of various genes in many basidiomycetes.

In *C. cinerea*, promoter analysis was conducted by monitoring gene expression in pools of 10 stable transformants for each mutant construct to overcome the differences in copy number and positional effect among individual transformants (Bertossa et al. 2004). However, the analysis of large numbers of transformants in other mushrooms with lower transformation efficiencies is difficult and laborious. In limited mushroom species, such as *S. commune* (de Jong et al. 2010), *C. cinerea* (Nakazawa et al. 2011), and *P. ostreatus* (Salame et al. 2012), the isolation of strains deficient in non-homologous DNA end joining permitted gene targeting. In these fungi, a gene targeting technique may be used in introduction of desired mutations in the promoter region on the chromosome, or to knock-in a reporter construct on a target site on the chromosome. However, it is not practical to isolate multiple strains with a series of mutant promoter sequences using gene targeting. In this context, the promoter assay system used in the present study offers simple and easy, initial screening of important sequences required for gene transcriptional regulation.

**Fig. 6:**
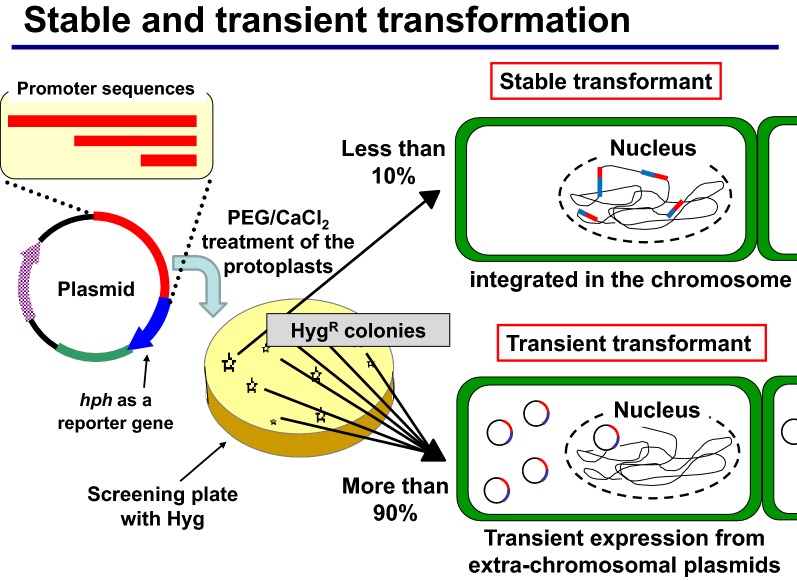
Schematic representation of stable and transient transformation. Introduced recombinant *hph* plasmids confer hygromycin resistance by direction of its promoter sequence. Transient transformants may express *hph* extrachromosomally and lose the plasmid during repeated transfer on the fresh screening plates

Generally, eukaryotic transcription occurs in the nucleus, then mRNA processing results in matured mRNA that is delivered and translated in the cytoplasm. Therefore, it is plausible that introduced DNA was delivered to the nucleus where transcription was directed by the promoter sequence of recombinant *hph* in the transient transformants of the present study (Fig. 6). The *hph* gene may be transcribed most provably in an extra-chromosomal manner like viral genes expressed after infection, although the possibility that the introduced sequence was once integrated on the host chromosome followed by precise excision after the transcription can not be completely ruled out. Upon cell division, the free plasmid DNA may be diluted during mycelial growth because of possible lack of replication and segregation functions. Suppose that delivery of the introduced DNA in the nucleus occurs as a prerequisite for chromosomal integration, then similar transient expression of introduced DNA may occur in stable transformation systems in other fungi. It may be possible to detect the transient expression of introduced DNA in the cell for a short time with a suitable selection system. In *C. cinerea*, Binninger et al. reported transforming-DNA-dependent unstable prototroph colonies in their transformation experiments using tryptophan auxotroph strain as a host and a plasmid harboring *TRP1* gene (1987), These unstable transformants were observed approximately in 100-fold number compared to stable transformants and initially grew to a limited extent on the first screening plate and described as ‘abortive’ transformants.

*hph* encodes hygromycin phosphotransferase and confers drug resistance to the host cell through detoxifying hygromycin B by phosphorylation (Punt and Hondel 1992). The extent of *hph* expression appears to determine whether the cell survives and grows to form a colony on medium containing a certain concentration of hygromycin. This property of the hygromycin resistant marker system may make it possible to observe colonies with unstable drug resistance by transient gene expression. We observed that high concentrations of salts, like MgSO_4_, affected tolerance of the host cell to hygromycin B during protoplast regeneration. Therefore, the balance between the transiently expressed hygromycin phosphotransferase and amount of hygromycin in the selection condition may be important to detect the transient transformation.

Another popular drug resistant marker gene, a carboxin resistant gene from *P. ostreatus* (Honda et al. 2000) encodes a mutant protein subunit of the mitochondrial enzyme complex succinate dehydrogenase (SDH) complex II, which is thought to be the target of the drug. We seldom observed unstable carboxin-resistant colonies in PEG/CaCl_2_-mediated transformation using pTM1 (Honda et al. 2000; Tsukihara et al. 2006). In this case, the drug resistance phenotype appeared to be an all-or-none situation. It is conceivable that the mutant protein acts as a bypass to avoid intracellular respiration inhibition by carboxin which enables survival in the presence of the drug. Moreover, drug resistance does not seem to extend to other cells without the mutant protein, which may result in difficulty for transient drug resistant transformants to grow and form a colony. The differences in mode of action by the two drug resistant markers may result in different tendencies of transient transformation. Additionally, the carboxin-resistant marker may be more species-specific, since the carboxin resistant marker from *P. ostreatus* does not function in other species (Irie et al. 2003; unpublished data). This may come from the disability of its gene product to associate and form an active complex with other endogenous subunits of the SDH complex II in the host. Therefore, it is recommended to make a homologous mutant *sdi1* to develop a carboxin-resistant marker system in each mushroom, as seen in *L. edodes* (Irie et al. 2003), *A. aegerita* (Herzog et al. 2019), *G. lucidum* (Xu et al. 2012), and *P. eryngii* (Shang et al. 2018).

In the present work, the number of hygromycin-resistant colonies on the regenerating plate was used as an index for gene expression of the introduced recombinant *hph*. It corresponds to the number of isolates that expressed hygromycin phosphotransferase over a survival threshold to enable colony formation in the presence of the drug, and thus it may be used to evaluate promoter activities in a certain range of the strength. In this work, it was demonstrated that the number of drug-resistant colonies can be used to determine the minimal basic *gpd* promoter region in *C. subvermispora*. We observed a significant difference in the number of hygromycin-resistant colonies between plasmids containing 141-bp and 120-bp promoter fragments (Fig. 4), suggesting that the 21-bp region between − 141 and − 120 contains essential sequences for promoter function. In silico analysis revealed that a GCN4 binding site (− 141 to 132) exists in this region, and experiments to elucidate the functional importance of this sequence are underway.

Generally, a TATA box plays an important role as the entry site for the preinitiation complex that directs start of transcription in many eukaryotic genes (Kinghorn and Turner 1992). In Basidiomycetes, genes contain a sequence similar to the TATA box in the promoter region have been reported, but their function has not been fully characterized (Kondoh and Shishido 1995; Kilaru and Kües 2005; Sakamoto et al. 2006; Itagaki et al. 2013). Our mutational analysis suggested that the TATAAA (− 63 to − 58) sequence plays an important role in the *C. subvermispora gpd* promoter. However, it is not clear whether the TATA sequence is essential for the function of the *Csgpd* promoter because we cannot rule out the possibility that the mutant sequence (TATCAA) retains a partial function of the TATA sequence or that there might be functional redundancy in the plasmid p201[A60C]. To precisely evaluate the amount of gene expression, it will be necessary to monitor the relationship between transcript accumulation and the extent of resistance to different concentrations of hygromycin. It is also plausible that other reporter genes, such as green fluorescent protein and luciferase, could be used as alternative indicators to quantitatively evaluate gene expression when combined with a gene knock-in. On the other hand, no TATA box was identified in the promoter region of the beta-tubulin gene in *C. subvermispora* (data not shown). cis elements required for the basic function of this TATA-less promoter were successfully characterized using the transient transformation assay and will be published elsewhere.

The promoter assay system developed in this work can be utilized for quick and easy assays of other gene expression control mechanisms, such as transcriptional termination, mRNA maturation, and translation efficiency etc. Furthermore, the transient expression of introduced DNA without chromosomal integration is suitable for tentative short-lived protein expression, such as that of Cas9 protein in the CRISPR/Cas9 genome editing system, because no carry over of heterologous DNA can be expected in the resulting strain.

## Data Availability

All data supporting the claims of this manuscript are presented and made available in this manuscript. The wildtype *C. subvermispora* strain (Fp-90031-sp (ATCC 90467) is available from ATCC, and the base plasmid used for experiments herein; pPHT1 (Cummings et al. 1999) was a kind gift of Professor Miriam E. Zolan, Department of Biology at Indiana University. Plasmid pLG-*hph* (Hirano et al. 2003) was kindly gifted by Dr. Sato, Iwate Biotechnology Institute, Iwate, presently, Kitami Institute of Technology, Hokkaido, Japan. Plasmids, pCsGi-*hph* and pCsbtubi-*hph*, are available through YH.

## Abbreviations

bp: basepairs (of DNA)
PCR: polymerase chain reaction
CRISPR/Cas9: clustered regularly interspaced palindromic repeats (CRISPR)/CRISPR-associated protein (Cas)9
PDA: potato dextrose agar
YMPG: yeast malt peptone glucose
MM buffer: (0.5 M mannitol, 25 mM maleic acid buffer, pH 5.5) buffer
MMC buffer: (0.5 M mannitol, 25 mM maleic acid buffer, pH 5.5, 12.5 mM CaCl_2_) buffer
PEG: polyethylene glycol
GTC buffer: (2 M glucitol, 10 mM Tris–HCl pH 7.5, 25 mM CaCl_2_) buffer
HygB: hygromycin B

## References

[CR1] Alic M, Kornegay JR, Pribnow D, Gold MH (1989). Transformation by complementation of an adenine auxotroph of the lignin-degrading basidiomycete *Phanerochaete chrysosporium*. Appl Environ Microbiol.

[CR2] Amirta R, Tanabe T, Watanabe T, Honda Y, Kuwahara M, Watanabe T (2006). Methane fermentation of Japanese cedar wood pretreated with a white rot fungus, *Ceriporiopsis subvermispora*. J Biotechnol.

[CR3] Bertossa RC, Kües U, Aebi M, Künzler M (2004). Promoter analysis of *cgl2*, a galectin encoding gene transcribed during fruiting body formation in *Coprinopsis cinerea (Coprinus cinereus*). Fung Genet Biol.

[CR4] Binninger DM, Skrzynia C, Pukkila PJ, Casselton LA (1987). DNA-mediated transformation of the basidiomycete *Coprinus cinereus*. EMBO J.

[CR5] Chandel AK, Gonçalves BC, Strap JL, da Silva SS (2015). Biodelignification of lignocellulose substrates: an intrinsic and sustainable pretreatment strategy for clean energy production. Crit Rev Biotechnol.

[CR6] Cummings WL, Celerin M, Crodian J, Bronick JK, Jolan ME (1999). Insertional mutagenesis in *Coprinus cinereus*: use of a dominant selectable marker to generate tagged, sporulation-defective mutants. Curr Genet.

[CR7] de Jong JF, Ohm RA, de Bekker C, Wösten HAB, Lugones LG (2010). Inactivation of *ku80* in the mushroom-forming fungus *Schizophyllum commune* increases the relative incidence of homologous recombination. FEMS Microbiol Lett.

[CR9] Fernandez-Fueyo E, Ruiz-Dueñas FJ, Ferreira P, Floudas D, Hibbett DS, Canessa P, Larrondo LF, James TY, Seelenfreund D, Lobos S, Polanco R, Tello M, Honda Y, Watanabe T, Watanabe T, Ryu JS, Kubicek CP, Schmoll M, Gaskell J, Hammel KE, St John FJ, Vanden Wymelenberg A, Sabat G, Splinter BonDurant S, Syed K, Yadav JS, Doddapaneni H, Subramanian V, Lavín JL, Oguiza JA, Perez G, Pisabarro AG, Ramirez L, Santoyo F, Master E, Coutinho PM, Henrissat B, Lombard V, Magnuson JK, Kües U, Hori C, Igarashi K, Samejima M, Held BW, Barry KW, LaButti KM, Lapidus A, Lindquist EA, Lucas SM, Riley R, Salamov AA, Hoffmeister D, Schwenk D, Hadar Y, Yarden O, de Vries RP, Wiebenga A, Stenlid J, Eastwood D, Grigoriev IV, Berka RM, Blanchette RA, Kersten P, Martinez AT, Vicuna R, Cullen D (2012). Comparative genomics of *Ceriporiopsis subvermispora* and *Phanerochaete chrysosporium* provide insight into selective ligninolysis. Proc Natl Acad Sci USA.

[CR10] Fitzgibbon FJ, Nigam P, Singh D, Marchant R (1995). Biological treatment of distillery waste for pollution-remediation. J Basic Microbiol.

[CR11] Gang L, Ruixue L, Liu Q, Wang Q, Chen M, Li B (2006). A highly efficient polyethylene glycol-mediated transformation method for mushrooms. FEMS Microbiol Lett.

[CR12] Gems DH, Johnstone IL, Clutterbuck AJ (1991). An autonomously replicating plasmid transforms *Aspergillus nidulans* at high frequency. Gene.

[CR13] Herzog RW, Singh NK, Schmidt C, Lemke PA (1995). Presence of a P1 bacteriophage sequence in transforming plasmids of *Pleurotus ostreatus*. Curr Genet.

[CR14] Herzog R, Solovyeva I, Bölker M, Lugones LG, Hennicke F (2019). Exploring molecular tools for transformation and gene expression in the cultivated edible mushroom *Agrocybe aegerit*a. Mol Genet Genom.

[CR15] Hirano T, Sato T, Yaegashi K, Enei H (2003). Efficient transformation of the edible basidiomycete *Lentinus edodes* with a vector using a glyceraldehyde-3-phosphate dehydrogenase promoter to hygromycin B resistance. Mol Gen Genet.

[CR16] Honda Y, Matsuyama T, Irie T, Watanabe T (2000). Carboxin resistance transformation of the homobasidiomycete fungus *Pleurotus ostreatus*. Curr Genet.

[CR17] Irie T, Honda Y, Hirano T, Sato T, Enei H, Watanabe T, Kuwahara M (2001). Stable transformation of *Pleurotus ostreatus* to hygromycin B resistance using *Lentinus edodes* GPD expression signals. Appl Microbiol Biotechnol.

[CR18] Irie T, Honda Y, Watanabe T, Kuwahara M (2001). Homologous expression of recombinant manganese peroxidase genes in ligninolytic fungus *Pleurotus ostreatus*. Appl Microbiol Biotechnol.

[CR19] Irie T, Sato T, Saito K, Honda Y, Watanabe T, Kuwahara M, Enei H (2003). Construction of a homologous selectable marker gene for *Lentinula edodes* transformation. Biosci Biotechnol Biochem.

[CR20] Itagaki T, Motoyoshi N, Kobayashi H, Ogawa Y, Hirose D, Inokuchi N (2013). Cloning and characterization of ribonuclease T2 gene (*RNHe30*) from the basidiomycete, *Hericium erinaceum*. Mycoscience.

[CR21] Itoh H, Wada M, Honda Y, Kuwahara M, Watanabe T (2003). Bioorganosolve pretreatments for simultaneous saccharification and fermentation of beech wood by ethanolysis and white rot fungi. J Biotechnol.

[CR22] Kilaru S, Kües U (2005). Comparison of *gpd* genes and their protein products in basidiomycetes. Fungal Genetics Newsletter.

[CR23] Kilaru S, Hoegger PJ, Majcherczyk A, Burns C, Shishido K, Bailey A, Foster GD, Kües U (2006). Expression of laccase gene *lcc1* in *Coprinopsis cinerea* under control of various basidiomycetous promoters. Appl Microbiol Biotechnol.

[CR24] Kim BG, Joh JH, Yoo YB, Magae Y (2003). Transformation of the edible basidiomycete *Pleurotus ostreatus* to phleomycin resistance. Mycobiology.

[CR25] Kinghorn JR, Turner G (1992). Applied molecular genetics of filamentous fungi.

[CR26] Kondoh O, Shishido K (1995). Characterization of the promoter region of a cell-adhesion protein gene derived from the basidiomycete *Lentinus edodes*. FEMS Microbiol Lett.

[CR27] Kües U (2015). Fungal enzymes for environmental management. Curr Opin Biotechnol.

[CR28] Kuo CY, Chou SY, Huang CT (2004). Cloning of glyceraldehyde-3-phosphate dehydrogenase gene and use of the *gpd* promoter for transformation in *Flammulina velutipes*. Appl Microbiol Biotechnol.

[CR29] Lubliner S, Regev I, Lotan-Pompan M, Edelheit S, Weinberger A, Segal E (2015). Core promoter sequence in yeast is a major determinant of expression level. Genome Res.

[CR30] Ma B, Mayfield MB, Gold MH (2003). Homologous expression of *Phanerochaete chrysosporium* manganese peroxidase, using bialaphos resistance as a dominant selectable marker. Curr Genet.

[CR31] Manubens A, Avila M, Canessa P, Vicuña R (2003). Differential regulation of genes encoding manganese peroxidase (MnP) in the basidiomycete *Ceriporiopsis subvermispora*. Curr Genet.

[CR32] Mellon FM, Casselton LA (1988). Transformation as a method of increasing gene copy number and gene expression in the basidiomycete fungus *Coprinus cinereus*. Curr Genet.

[CR33] Messner K, Srebotnik E (1994). Biopulping: an overview of developments in an environmentally safe paper-making technology. FEMS Microbiol Rev.

[CR34] Munoz-Rivas A, Specht AC, Drummond BJ, Froeliger E, Novotny CP, Ullrich RC (1986). Transformation of the basidiomycetes, *Schizophyllum commune*. Mol Gen Genet.

[CR35] Nakazawa T, Ando Y, Kitaaki K, Nakahori K, Kamada T (2011). Efficient gene targeting in ∆*Cc.ku70* or ∆*Cc.lig4* mutants of the agaricomycete *Coprinopsis cinerea*. Fung Genet Biol.

[CR36] Nakazawa T, Izuno A, Horii M, Kodera R, Nishimura H, Hirayama Y, Tsunematsu Y, Miyazaki Y, Awano T, Muraguchi H, Watanabe K, Sakamoto M, Takabe K, Watanabe T, Isagi Y, Honda Y (2017). Effects of *pex1* disruption on wood lignin biodegradation, fruiting development and the utilization of carbon sources in the white-rot Agaricomycete *Pleurotus ostreatus* and non-wood decaying *Coprinopsis cinerea*. Fungal Genet Biol.

[CR37] Nayan N, Sonnenberg ASM, Hendriks WH, Cone JW (2018). Screening of white-rot fungi for bioprocessing of wheat straw into ruminant feed. J Appl Microbiol.

[CR38] Nishimura H, Sasaki M, Seike H, Nakamura M, Watanabe T (2012). Alkadienyl and alkenyl itaconic acids (ceriporic acids G and H) from the selective white-rot fungus *Ceriporiopsis subvermispora*: a new class of metabolites initiating ligninolytic lipid peroxidation. Org Biomol Chem.

[CR39] Ohashi Y, Kan Y, Watanabe T, Honda Y, Watanabe T (2007). Redox silencing of the Fenton reaction system by an alkylitaconic acid, ceriporic acid B produced by a selective lignin-degrading fungus, *Ceriporiopsis subvermispora*. Org Biomol Chem.

[CR40] Okano K, Kitagawa M, Sasaki Y, Watanabe T (2005). Conversion of Japanese red cedar (*Cryptomeria japonica*) into feed for ruminants by white-rot basidiomycetes. Anim Feed Sci Technol.

[CR41] Otjen L, Blanchette R, Effland M, Leatham G (1987). Assessment of 30 white rot basidiomycetes for selective lignin degradation. Holzforschung.

[CR42] Peng M, Singh NK, Lemke PA (1992). Recovery of recombinant plasmids from *Pleurotus ostreatus* transformants. Curr Genet.

[CR43] Punt PJ, Hondel CAMJJVD (1992). Transformation of filamentous fungi based on hygromycin B and phleomycin resistance markers. Meth in Enzymol.

[CR44] Punt PJ, Dingemanse MA, Kuyvenhoven A, Soede RDM, Pouwels PH, Hondel CAMJJVD (1990). Functional elements in the promoter region of *Aspergillus nidulans gpdA* gene encoding glyceraldehyde-3-phosphate dehydrogenase. Gene.

[CR45] Randall TA, Reddy CA (1992). The nature of extrachromosomal maintenance of transforming plasmids in the filamentous basidiomycete *Phanerochaete chrysosporium*. Curr Genet.

[CR46] Sakamoto Y, Watanabe H, Nagai M, Nakade K, Takahashi M, Sato T (2006). *Lentinula edodes tlg1* encodes a thaumatin-like protein that is involved in lentinan degradation and fruiting body senescence. Plant Physiol.

[CR47] Salame T, Knop D, Tal DT, Levinson D, Yarden O, Hadar Y (2012). Predominance of a versatile-peroxidase-encoding gene, *mnp4*, as demonstrated by gene replacement via a gene targeting system for *Pleurotus ostreatus*. Appl Environ Microbiol.

[CR48] Sasaki C, Takada R, Watanabe T, Honda Y, Karita S, Nakamura Y, Watanabe T (2011). Surface carbohydrate analysis and bioethanol production of sugarcane bagasse pretreated with the white rot fungus, *Ceriporiopsis subvermispora* and microwave hydrothermolysis. Bioresour Technol.

[CR49] Sato T, Yaegashi K, Ishii S, Hirano T, Kajiwara S, Shishido K, Enei H (1998). Transformation of the edible basidiomycetes *Lentinus edodes* by restriction enzyme-mediated integration of plasmid DNA. Biosci Biotechnol Biochem.

[CR50] Sawada T, Nakamura Y, Kobayashi F, Kuwahara M, Watanabe T (1995). Effects of fungal pretreatment and steam explosion pretreatment on enzymatic saccharification of plant biomass. Biotechnol Bioeng.

[CR51] Schillberg S, Tiburzy R, Fischer R (2000). Transient transformation of the rust fungus *Puccinia graminis* f. sp. *tritici*. Mol Gen Genet.

[CR52] Shang J, Li Y, Yang R, Wang Y, Mao W, Tang L, Wu Y, Nakazawa T, Honda Y, Li Y, Bao D (2018). Efficient transformation of *Pleurotus eryngii* with a safe selective marker mutated from the *Pesdi1* gene. J Microbiol Meth.

[CR53] Shi T, Liu G, Ji R, Shi K, Song P, Ren L, Huang H, Ji X (2017). CRISPR/Cas9-based genome editing of the filamentous fungi: the state of the art. Appl Microbiol Biotechnol.

[CR54] Sun L, Cai H, Xu W, Hu Y, Gao Y, Lin Z (2001). Efficient transformation of the medicinal mushroom *Ganoderma lucidum*. Plant Mol Biol Reporter.

[CR55] Tello M, Corsini G, Larrondo LF, Salas L, Lobos S, Vicuña R (2000). Characterization of three new manganese peroxidase genes from the ligninolytic basidiomycete *Ceriporiopsis subvermispora*. Biochim Biophys Acta.

[CR56] Treu R, Falandysz J (2017). Mycoremediation of hydrocarbons with basidiomycetes-a review. J Environ Sci Health B.

[CR57] Tsukihara T, Honda Y, Watanabe T, Watanabe T (2006). Molecular breeding of white rot fungus *Pleurotus ostreatus* by homologous expression of its versatile peroxidase MnP2. Appl Microbiol Biotechnol.

[CR58] Tsukihara T, Honda Y, Sakai R, Watanabe T, Watanabe T (2008). Mechanism for oxidation of high-molecular-weight substrates by a fungal versatile peroxidase, MnP2. Appl Environ Microbiol.

[CR59] Urzúa U, Kersten PJ, Vicuña R (1998). Kinetics of Mn^3+^-oxalate formation and decay in reactions catalyzed by manganese peroxidase of *Ceriporiopsis subvermispora*. Arch Biochem Biophys.

[CR60] van de Rhee MD, Graca PMA, Huzing HJ, Mooibroek H (1996). Transformation of the cultivated mushroom, *Agaricus bisporus* to hygromycin B resistance. Mol Gen Genet.

[CR61] Wall MB, Cameron DC, Lightfoot EN (1993). Biopulping process design and kinetics. Biotechnol Adv.

[CR62] Watanabe T, Teranishi H, Honda Y, Kuwahara M (2002). A selective lignin-degrading fungus, *Ceriporiopsis subvermispora*, produces alkylitaconates that inhibit the production of a cellulolytic active oxygen species, hydroxyl radical in the presence of iron and H_2_O_2_. Biochem Biophys Res Commun.

[CR63] Xu JW, Xu YN, Zhong JJ (2012). Enhancement of ganoderic acid accumulation by overexpression of an N-terminally truncated 3-hydroxy-3-methylglutaryl coenzyme A reductase gene in the basidiomycete *Ganoderma lucidum*. Appl Environ Microbiol.

[CR64] Yanisch-Perron C, Vieira J, Messing J (1985). Improved M13 phage cloning vectors and host strains: nucleotide sequences of the M13mp18 and pUC19 vectors. Gene.

[CR65] Yano S, Yamamoto S, Toge T, Wakayama M, Tachiki T (2003). Occurrence of a specific protein in basidiomycete-lytic enzyme preparation produced by *Bacillus circulans* KA-304 inductively with a cell-wall preparation of *Schizophyllum commune*. Biosci Biotechnol Biochem.

